# Chilblains

**Published:** 1874-05

**Authors:** 


### CHILBLAINS

are caused by putting the feet too close to the fire. They become excessively troublesome and are sometimes difficult of cure.

Dissolve six drachms of tannin in two pints of water, then dissolve forty grains of iodine in two ounces of alcohol; mix all together and add enough water to make two quarts, to be used every night thus: Pour the mixture into an earthenware or porcelain dish; hold it over the fire, and hold the affected part in the liquid until it becomes so hot that it cannot be comfortably borne any longer, then hold the affected part to the fire until perfectly easy.

The first application gives prompt relief, to be continued until cured.

The same liquid can be used every time.

